# Whole-genome analysis of a *Vibrio cholerae* O1 biotype classical strain isolated in 1946 in Sasebo city, Nagasaki prefecture, from a returnee from the northeast part of China

**DOI:** 10.1186/s41182-023-00500-4

**Published:** 2023-02-02

**Authors:** Ken-Ichiro Kuninobu, Taichiro Takemura, Yu Takizawa, Futoshi Hasebe, Tetsu Yamashiro

**Affiliations:** 1 Graduate School of Biomedical Sciences, Nagasaki University, 1-12-4 Sakamoto Nagasaki city, Nagasaki, 852-8523 Japan; 2 Vietnam Research Station, Institute of Tropical Medicine, Nagasaki University, 1-12-4 Sakamoto Nagasaki city, Nagasaki, 852-8523 Japan; 3 Department of Bacteriology, Graduate School of Medicine, University of the Ryukyus, 207 Uehara Nishihara, Okinawa, 903-0215 Japan

**Keywords:** *Vibrio cholerae* O1, Biotype classical, Whole-genome analysis, Interpandemic between the 6th and the 7th cholera pandemic, De novo assembly, Phylogenetic tree, CTX prophage region, Sasebo city, Nagasaki prefecture

## Abstract

**Background:**

Cholera is a water-borne disease caused by toxigenic *Vibrio cholerae* serogroups O1 and O139. Not a few studies on the whole-genome analyses of *V. cholerae* O1 biotype El Tor have been published; however, the number of analyses for biotype classical is limited. The whole-genome analysis was made on a *V. cholerae* biotype classical strain, Man9, isolated in 1946 in Sasebo city, Nagasaki prefecture, from a returnee from the northeast part of China.

**Methods:**

PacBio RSII was used to determine the whole-genome of Man9. De novo assemblies were made with CLC Genomics Workbench 8.5.1 and Canu. 2.0 and annotated by Prokka version 1.12. Upon determining the configuration of the CTX prophage region, combined procedures of PCR, RFLP with Southern blotting, and Sanger sequencing method were used. The phylogenetic tree was constructed by RaxML and visualized by Phandango. The identification of Cas genes and spacer sequences was made by CRISPR-finder and NCBI Blast search. These data were compared with those of *V. cholerae* serogroup O1 biotype classical O395.

**Results:**

The Man9 carried the 2.9 Mb (Chr1) and 1.1 Mb (Chr2) chromosomes with 2683 and 1198 CDSs, respectively. The genome similarity between Man9 and O395 was 97.0% when the total genomes were compared. Man9 carried a 380-kb inversion on the Chr1, and 95-kb and 35-kb fragments were not present on the Chr1 and on the Chr2, respectively. Man9 monophyletically clustered with 23 other biotype classical strains on the core gene phylogenetic tree analyses. Man9 carries “CTX^cla^” and a stretch of “truncated CTX^cla^-CTX^cla^” on the Chr1 and the Chr2, respectively, which is the opposite arrangement of O395. Man9 carries CRISPR–Cas system subtype I-E with 33 spacers, 64% of which were identical to those of O395.

**Conclusions:**

Man9 differs from O395 by 3% on the total genome comparison; however, genomic analysis of a strain having circulated in the interpandemic period between the 6th and the 7th cholera pandemic is valuable and contributes to understanding the evolution of pathogenic *V. cholerae*.

**Supplementary Information:**

The online version contains supplementary material available at 10.1186/s41182-023-00500-4.

## Background

Cholera is a water-borne disease caused by *Vibrio cholerae* serogroups O1 and O139, which carry the genes for the cholera toxin responsible for acute watery diarrhea [1]. Toxigenic *V. cholerae* transmits by the fecal–oral route, in which bacteria are excreted in the diarrheal stool and are ingested by a vulnerable human to cause disease [2]. More than 10^7^ cfu/ml organisms are contained in stool, and the total output of up to 10–15 L per day causes environmental contamination and becomes a resultant source of infection [3]. A cholera outbreak is closely linked to inadequate access to clean water and sanitation facilities. According to the World Health Organization, it is estimated that 1.3–4 million cases with 21,000–143,000 deaths annually [4]. The disease has the unusual ability to spread rapidly to large numbers of people. A cholera epidemic can occur either in endemic areas or in areas where cholera does not regularly occur. In endemic populations, the incidence of cholera is greatest in children under 5 years, probably due to lower preexisting immunity to cholera. Contrary to endemic areas, incidence rates of cholera are age independent in the populations who have not had repeated exposure to cholera in the past [5].

Seven large-scale cholera pandemics have been recorded since the nineteenth century in the world. It is believed that the biotype classical *V. cholerae* serogroup O1 caused the first six cholera pandemics, but biotype El Tor emerged in 1961, spread globally, and replaced the biotype classical to cause the current 7th pandemic [1, 6]. Hu et al. proposed a hypothesis that the clonal transition from biotype classical to El Tor in a period between the 6th and the 7th pandemics. They revealed that the six-step evolution of the 7th pandemic strain from its probable origin in South Asia to its nonpathogenic form in the Middle East in ~ 1908 (stage 1, 2, 3) to Indonesia in ~ 1925 (stage 4), where it evolved into a pandemic strain (stage 5, 6) before becoming widespread in 1961 [7].

In Japan, it is reported that although severe cholera epidemics had occurred early in the twentieth century, the disease had almost completely disappeared before World War II. In 1946 however, the arrival of repatriation ships from China and other countries of the Far East led to the importation of cholera through ports and caused an epidemic reaching a total of 1229 cholera patients with 528 casualties [8]. Man9 is an isolate from a returnee from the northeast part of China in 1946 in Sasebo city, Nagasaki prefecture Japan. It is regarded as one of the causative agents of the biggest and the latest cholera epidemic in 100 years since 1922 in Japan. The year 1946 lies in the interpandemic period (1926–1960) between the 6th and the 7th cholera pandemic when biotype classical strains and various types of the pre-7^th^ pandemic strains were co-circulated [9]. However, genomic information on the strains circulating during the period is limited.

## Materials and methods

### *Vibrio cholerae* strains

The strain Man9 was isolated from a patient with severe diarrhea in Sasebo city, Nagasaki prefecture, Japan, in 1946, the following year World War II had ended. Sasebo city was appointed as one of the ports for receiving returnees from a war area. More than 1.3 million returnees had been transported to the Sasebo port in 1945–1950; of those, approx. 520,000 (40%) were returnees from the northeast part of China [10]. The patient from whom Man9 was isolated was likely a returnee from the northeast part of China because “Man”, an abbreviation of “Manchuria” which was once used to refer to the northeast part of China, was used as the identification code.

The strain Man9 had been stored with glycerol in a deep freezer with occasional passages in the Institute of Tropical Medicine, Nagasaki University. The strain was confirmed as *V. cholerae* serogroup O1, serotype Inaba, by biochemical and serological analyses using API 20 E kit (Biomeriuex, France), and *V. cholerae* antisera set (DENKA Co. Ltd., Tokyo Japan), respectively. Additionally, DNA sequence profiles of *tcpA, rstR,* and *ctxB* genes were identical to those of strain O395, a representative biotype classical strain.

### PacBio sequencing and assembly

The whole-genome sequence of Man9 was determined by PacBio RS II sequencing services provided by Takara Co ltd. In brief, ten micrograms of genomic DNA were fragmented into 10-kb inserts using Covaris g-Tubes (Covaris Inc., USA). The DNA library was prepared using a PacBio SMRTbell template preparation kit version 1.0. A SMRTbell template library was quantified, and the sheared DNA quality was checked with a Bioanalyzer DNA 12,000 chip (Agilent Technologies, USA). The sequencing of two SMRT cells with 180 min movie times was performed on a PacBio RS II system using P5 polymerase and C3 sequencing chemistry. The resulting short-length reads (< 200 bp) were filtered out with a home-customized script. De novo assemblies were carried out using default parameters in CLC Genomics Workbench 8.5.1, and Canu 2.0. The whole-genome data for Man9 were deposited to DDBJ with the accession numbers AP026558-AP026559.

### Genome annotation, phylogenetic tree and comparative genomic analyses

The circular genome map with the GC skew and the GC content was constructed by CGView Server software with default parameters [11]. Completed genome sequences of the Chr1 and the Chr2 of Man9 were annotated by Prokka version 1.12 [12]. Dot-plot analysis to compare the Man9 genome with that of O395 (Chr1, CP000627.1; Chr2, CP000626.1) was performed using the GenomeMatcher software [13].

The pangenome analysis was performed with the Roary version 3.11.2 at the default setting [14] on a total of 35 genome sequences of *V. cholerae* O1 strains (24 biotype classical; 11 7th pandemic biotype El Tor) and one genome sequence of *V. cholerae* non O1, non O139 strain listed in the Additional file 2: Table S1. The phylogenetic tree based on the SNPs in the core genes was constructed by RaxML8.2.10 [15] and visualized using Phandango 1.3.0 [16]. The biological functions of identified unique genes on either Man9 or O395 were analyzed by the KEGG pathway database [17]. For the CRISPR–Cas array, the identification of Cas genes and spacer sequences was made by CRISPR-finder [18]. The target of each spacer was analyzed by the NCBI Blast search.

### Confirmation of the configuration of the CTX prophage regions

Man9 and O395 were examined by PCR with a series of primers [19] to confirm the information on the presence or the absence of the CTX prophage-associated genes and their positional relationships on the Chr1 and the Chr2 [20]. To estimate the configuration of the CTX prophage regions spanning from the TLC gene clusters [21] to the RTX gene clusters [22], RFLP with Southern blotting was performed, as described elsewhere [20, 23]. Fragments comprising the CTX prophage regions were amplified from the bacterial genome by PCR using a set of primer pairs and protocols described in the previous study [20] and sequenced.

## Results

### Construction of Man9 genome and its comparative analysis with O395

**Fig. 1:**
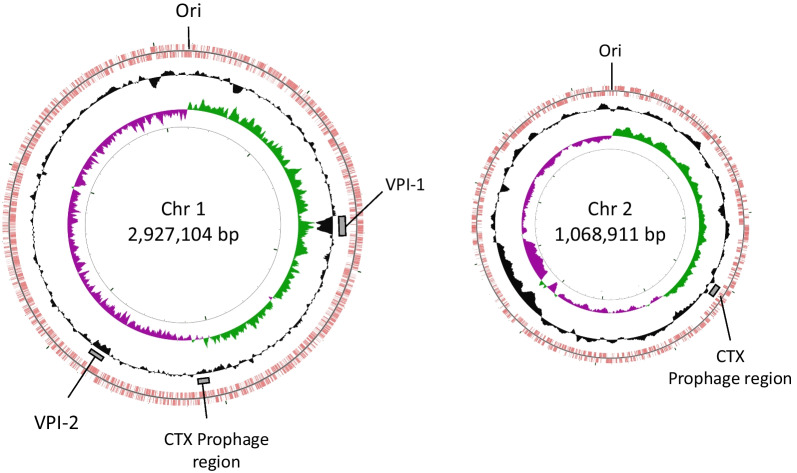
Circular representation of the two chromosomes of *Vibrio cholerae* O1 biotype classical strain Man9 is illustrated. The outermost circle and the 2nd circle indicate the positions of the putative protein-encoding genes in clockwise and counter-clockwise directions, respectively. The 3rd circle represents the GC content which shows the deviation from the average GC content of the entire sequence. The 4th circle represents the GC skew. *Chr* chromosome, *Ori* replication origin, *VPI* Vibrio pathogenic island

The complete de novo Man9 genome was constructed in the study. The assembly of the Man9 genome with long-read data was fully resolved in two large contigs corresponding to the large (Chr1) and the small (Chr2) chromosome, with an average coverage depth of 276 × in total, the number of reads of 211,140 and the average read length of 5224 bp. The constructed genome of Man9 was analyzed and compared with that of O395 by NCBI Blast search. The size of the Chr1 and the Chr2 of Man9 was determined as 2,927,104 bp, and 1,068,911 bp, respectively (Fig. 1). The Prokka pipeline annotation indicated that Man9 was estimated to contain 2683 and 1198 possible CDS on the Chr1 and the Chr2, respectively, which is comparable to those of O395 (Chr1, 2692 CDS; Chr2, 1042 CDS) [24].

The average GC content of the Man9 genome was approx. 47.5% and several lower-GC regions were contained (Fig. 1). Man9 was determined to carry representative major virulence regions, including the CTX prophage region, the vibrio pathogenic islands 1 (VPI-1) and 2 (VPI-2) in 1,025,101–1,031,422 bp; 347,741–388,429 bp; and 1,328,482–1,392,061 bp, respectively, on the Chr1; and another set of the CTX prophage region in 560,243–568,607 bp on the Chr2 (Fig. 1).

**Fig. 2:**
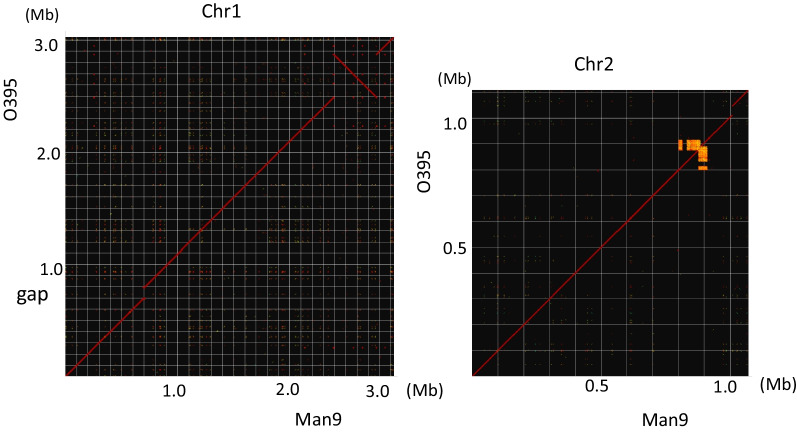
Dot-plot analysis of genomes of Man9 and O395. Gridlines are drawn every 100 kb. Note that fragments not present in Man9 were shown as gaps at approx. 0.7 and 1.1 Mb on the Chr1 and the Chr2, respectively. Approx. 380 kb inversion is present at approx. 2.4 Mb on the Chr1 of Man9. Chr, chromosome

The dot-plot analysis between Man9 and O395 indicated that a 380,927-bp fragment was inversely present at 2,388,702 bp on the Chr1 of Man9 (Fig. 2, Chr1). Three hundred and sixty-four (364) CDSs were estimated to be associated with metabolisms, nucleic acid synthesis, structures, transcriptions, clustered regulatory interspaced short palindromic repeats (CRISPR); CRISPR associated (Cas) protein system, exogenous phages, and drug resistance (data not shown). Suspected superintegron regions were found at 80–90 kb on the Chr2 of the strains (Fig. 2, Chr2).

Man9 lacks a 94,615-bp fragment which is present at 700,628 bp on the Chr1 of O395, and it is depicted as a gap in Fig. 2 (Chr1). The fragment not present in Man9 was analyzed to contain 95 CDSs associated with exogenous phages (22 CDSs, 23.1%), bacterial metabolisms (29 CDSs, 30.5%), and bacterial structures (5 CDSs, 5.3%) (Additional file 1: Fig. S1). On the Chr2, a 35-kb gap was observed at approx. 1.1 Mb, indicating that the fragment containing 46 CDS was not present in the Chr2 of Man9 (Fig. 2, Chr2). The total DNA sequence similarity between Man9 and O395 was 97.0% when the 94 kb and the 35 kb fragments were considered and 99.9% when not considered.

Comparative genomic analysis on Man9 and O395 was made with the Kyoto Encyclopedia of Genes and Genomes (KEGG) method to identify unique CDSs carried by Man9 and O395. Of the total 3881 CDSs in Man9, 35 CDSs were unique to Man9, which were carried exclusively on the Chr2 (Additional file 1: Fig. S2). Of the 35 CDSs unique to Man9, three and two were associated with bacterial metabolisms and environmental information processing, respectively, leaving the remaining 30 CDSs unknown (Additional file 1: Fig. S2). When the analysis was made based on the O395 genome, of the total 3734 CDSs (2,692 on the Chr1 and 1042 on the Chr2), 124 CDSs were unique to O395, of those 69 and 55 were on the Chr1 and the Chr2, respectively (Additional file 1: Fig. S2). All the 69 and 46 out of the 55 CDS were present in the 94-kb and the 35-kb fragments, respectively, both of which were not present in Man9.

### Phylogenetic tree

**Fig. 3:**
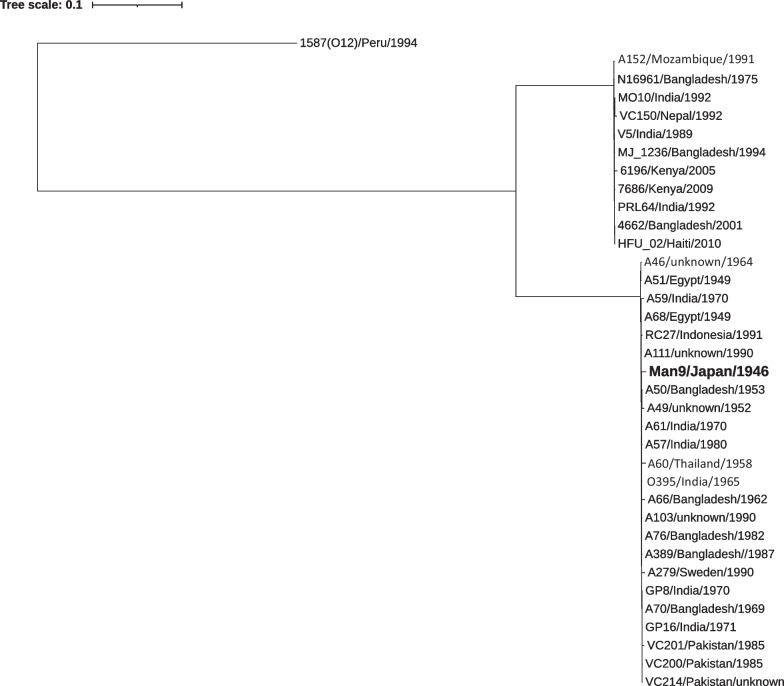
The maximum likelihood phylogenetic tree of the 24 *V. cholerae* O1 biotype classical, 11 7th pandemic biotype El Tor strains, and one *V. cholerae* non O1, non O139 strain based on the SNP differences across the whole core genomes

A phylogenetic tree was constructed using 24 *V. cholerae* O1 biotype classical strains, including Man9, 11 7th pandemic biotype El Tor strains, and one *V. cholerae* non O1, non O139 strain (1587, serogroup O12) (Additional file 2: Table S1). Man9, along with 23 other biotype classical strains, formed a monophyletic cluster independent of that formed by biotype El Tor strains (Fig. 3).

### The composition and configuration of the CTX prophage region of Man9

The PCR profiles of Man9 and O395 genomic DNA revealed identical to each other (Additional file 3: Table S2), indicating that the genetic components of the CTX prophage regions of Man9 were close to those of O395. When the Man9 genome was digested with *BglI*, four fragments (13, 11.2, 5.8, and 3.5 kb) and two fragments (11.2 and 3.5 kb) were visualized when *zot*- and *ctxA*-specific probes, respectively, were used (Additional file 3: Table S2 and Additional file 1: Fig. S3). The analysis indicated that Man9 carried a single copy of the biotype classical specific-CTX prophage (CTX^cla^) region harboring the classical specific *rstR* (*rstR*^*cla*^) and *ctxB* (*ctxB*^*cla*^) on the Chr1 (Additional file 1: Fig. S4). Meanwhile, a truncated form of the CTX^cla^ (CTX^cla^Trunc.) harboring *rstR*^*cla*^, *rstA*, *rstB*, and a 24 bp sized fragment (X) in this order, followed by a single copy of the complete CTX^cla^ (CTX^cla^Trunc.-CTX^cla^) was carried on the Chr2 (Additional file 1: Fig. S4). These CTX region configurations either on the Chr1 or Chr2 were consistent with data obtained from the PacBio RSII, RFLP by Southern blotting, and PCR profile. Similar procedures were applied to O395 and confirmed that the configuration of the CTX prophage region was consistent with those deposited in the database, that is, the CTX^cla^Trunc.-CTX^cla^ on the Chr1 and the CTX^cla^ on Chr2, which was the opposite arrangement to Man9.

### CRISPR–Cas system

**Fig. 4:**
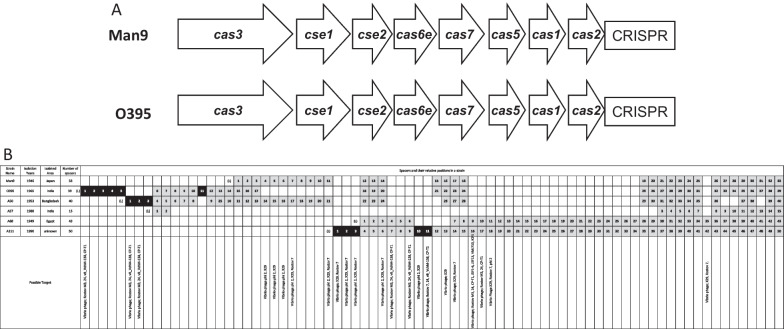
**A** Operon alignments associated with the CRISPR–Cas system carried by Man9 or O395 are shown. The system is classified as subtype I-E. **B** Spacer alignments of CRISPR–Cas in six *V. cholerae* biotype classical strains, including Man9, are shown. Spacers are represented as numbered rectangles. Arrays are oriented with respect to the leader sequence located at the left. Black rectangles represent unique spacers present in the strain indicated. Identical spacers shared with more than one strain are shown with gray rectangles and aligned vertically. Missing spacers are indicated as blank. Possible known targets are listed at the bottom of the figure. *(L)* leader sequence

Man9 was identified to carry the 2041-bp CRISPR–Cas system with 33 spacers in the 380,927-bp inversion. The system contained a Cas protein gene cluster, *cas3*-*cse1*-*cse2*-*cas6e*-*cas7*-*cas5*-*cas1*-*cas2*, followed by a CRISPR array (Fig. 4A) with 28-bp canonical repeat and 33 spacers. This is a variant Cas gene arrangement for the subtype I-E system [25], which was identical to that carried by O395 (Fig. 4A). Of the 33 Man9 spacers, 25 were shared by O395, which was 64.1% (25/39) of all spacers carried by O395 (Fig. 4B) (spacer coincidence ratio). All 33 Man9 spacers were shared also by four other biotype classical strains with varying spacer coincidence ratio from 44.0% (22/50; strain A111) to 86.7% (13/15; strain A57) (Fig. 4B). Eight out of the 33 spacers of the Man9 were estimated to target respective overlap regions, accounting “Vibrio phage phi2 and X29” for 3 spacers; “Vibrio phage phi2, X29, and Rostov7” for 3 spacers; “Vibrio phage and X29” for 1 spacer; and “Vibrio phage, X29, and Rostov7” for 1 spacer (Fig. 4B).

## Discussion

With the development of the next-generation sequencing technologies, not a few studies on the whole-genome analyses of *V. cholerae* O1 biotype El Tor strains have been published [26–28]; however, reports for biotype classical which have been playing the principal role in the 1st–6th cholera pandemics are not enough because the number of biotype classical strains in storage is limited. The present study describes the whole-genome analyses of *V*. *cholerae* O1 biotype classical Man9, isolated from a returnee from the northeast part of China at the end of World War II to Sasebo city, Nagasaki prefecture in 1946.

The genetic background of Man9 was very close to that of O395 with 97.0% similarity when the total genome was compared; however, several differences were noted. One of those was the presence of approx. 380 kb inversion on the Chr1 of Man9 (Fig. 2, Chr1). Several transposons or the presence of repeated sequences at both ends of the region sometimes induce inversions [29, 30]; however, these elements were not found in the proximity of the fragment. In addition, the 95 kb and the 35 kb fragments present on the Chr1, and the Chr2, respectively, of O395 were not found in Man9 (Fig. 2). KEGG analysis for searching unique CDSs in Man9 or O395 was made and identified 35 and 124 CDSs unique to Man9, and O395, respectively. Man9 carries the unique CDSs exclusively on the Chr2, whereas most of those in O395 were carried in the 95-kb and the 35-kb fragments.

Mutreja et al. analyzed the genetic backgrounds of the 7th pandemic biotype El Tor using more than 100 isolates in the 1957–2010 period and concluded that they were genetically monophyletic, differing only 50–250 SNPs from the representative 7th pandemic biotype El Tor strain N16961 isolated in 1975 [28]. They also indicated that the *V. cholerae* O1 biotype classical isolates formed a distinct and highly clustered group from the 7th pandemic biotype El Tor isolates. In the present study, the 24 biotype classical strains including Man9, the isolation years of those ranges in 1946–1991, were almost monophyletic in the genetic backgrounds (Fig. 3).

Pham et al. reported that the configurations of the CTX prophage region of the 7th pandemic biotype El Tor wave 1 strains were quite diverse [20]. In the present study, Man9 was identified to carry the CTX prophage region containing a single copy of CTX^cla^ and a stretch of CTX^cla^ Trunc.-CTX^cla^ on the Chr1 and the Chr2, respectively (Additional file 1: Fig. S4), which is the opposite arrangement of O395. It is necessary to analyze the CTX prophage regions carried by biotype classical strains to see whether they are as diverse as those of biotype El Tor strains using a large number of strains.

The CRISPR–Cas system is the adaptive antivirus immunity system carried by many bacteria [31]. The system is ubiquitous in strains of the biotype classical but absent in El Tor [32]. Similar to O395, the CRISPR–Cas system carried in Man9 was identified to be subtype I-E, as it contained *cas3*, *cse1,* and *cse2* genes, the signature genes of the subtype (Fig. 4A) [25]. It seemed reasonable that all the 33 CRISPR–Cas spacers carried by Man9 were shared with any of the five biotype classical strains compared, as Man9 had been circulated earlier period than the other strains (Fig. 4B).

*V. cholerae* O1 biotype classical Man9 was isolated in 1946. The year corresponds to the interpandemic period between the 6th (1899–1923) and the 7th (1961 ongoing) cholera pandemic. In this period, several cholera epidemics occurred but were confined locally to South Asia, Southeast Asia, and the Far East and did not develop into pandemics [6]. At that time, biotype classical strains and different types of the pre-7th pandemic strains were co-circulated as possible causative agents [9]. During the period, China suffered cholera epidemics severely in 1940 and 1946 [6], and a lineage of Man9 is likely the causative agent, and information on the strains circulating in the period is limited. Data on the whole-genome analysis of Man9 and its genomic comparison with O395 would provide valuable information to fill in the missing pieces and contribute to understanding the evolution of pathogenic *V. cholerae*.

## Conclusions

Whole-genome analysis was made on a *V. cholerae* O1 biotype classical strain Man9, isolated from a returnee from the northeast part of China to Sasebo city, Nagasaki prefecture Japan, in 1946. The Man9 carried the 2.9 and 1.1 Mb chromosomes with 2683 and 1198 CDSs, respectively. The genome similarity between Man9 and O395 was 97.0% when the total genome was compared. Genomic comparison between Man9 and O395 revealed that Man9 carried a 380-kb inversion on the Chr1, and the 95-kb and the 35-kb fragments carried by O395 were not present on the Chr1 and the Chr2, respectively. Man9 monophyletically clustered with 23 other biotype classical strains on the core gene phylogenetic tree analyses. Man9 carries CTX^cla^ and a stretch of truncated CTX^cla^- CTX^cla^ on the Chr1 and the Chr2, respectively, which is the opposite arrangement of O395. Man9 carries CRISPR–Cas system subtype I-E with 33 spacers, 25 of which were identical to those of O395. Man9 circulated during the interpandemic period between the 6th and the 7th cholera pandemics. Data on the whole-genome analysis of Man9 and its genomic comparison with O395 would provide valuable information and contribute to understanding the evolution of pathogenic *V. cholerae*.

## Supplementary Information

**Additional file 1: Fig. S1.** The 94,615 bp fragment present in *V. cholerae* O1 biotype classical O395 but not in the Man 9 genome is depicted. Ninety-five CDSs are present in the fragment. **Fig. S2**. Kyoto Encyclopedia of Genes and Genomes (KEGG) analyses on the unique CDSs in two chromosomes carried by each strain Man9 and O395 were made. Thirty-five CDSs are unique to Man9, whereas 124 CDSs are unique to O395. **Fig. S3.** Profile of the Restriction Fragment Length Polymorphism (RFLP) with Southern blotting. The genome of O395 (Lane 1) and Man9 (Lane 2) were digested with *BglI* and visualized with *zot*- and *ctxA*—specific probes. *M* molecular marker. **Fig. S4.** PCR with the primer pair of TLC3F plus RTX5R and CIIF plus CIIR was applied on the genomic DNA extracted from Man9. An approx. 7.5 and 9.8 kb fragments were produced in separate reactions, estimated to contain the CTX prophage region on the Chr1, and the Chr2, respectively. The 9.8 kb product was further fragmented into three fragments; a 2.7 kb fragment with CIIF and rstBR primers; a 1 kb fragment with rstBF and rstRR primers; and a 6.6 kb fragment with rstRF and CIIR primers by PCR. The best-estimated configuration of the CTX prophage region, a CTX^cla^ on the Chr1 and a CTX^cla^Trunc-CTX^cla^ stretch on the Chr2, carried by *Vibrio cholerae* O1 biotype classical strain Man9 is shown.

**Additional file 2: Table S1.** The List of *V. cholerae* O1 biotype classical strains (24 strains), biotype El Tor strains (11 strains), and a *V. cholerae* non O1, non O139 strain used to construct a phylogenetic tree.

**Additional file 3: Table S2.** The presence or the absence of the CTX prophage-associated genes and their positional relationship on the Chr1 and the Chr2 was confirmed by the PCR profile using a series of primers [19].

## Data Availability

The datasets used and analyzed during the present study are available from the corresponding author Professor Tetsu Yamashiro on reasonable request. The whole-genome data for Man9 were deposited to DDBJ with the accession numbers AP026558–AP026559.

## Abbreviations

Chr1: Chromosome 1
Chr2: Chromosome 2
CTX: Cholera toxin
RFLP: Restriction fragment length polymorphism
SNP: Single nucleotide polymorphism
CRISPR: Clustered Regularly Interspaced Short Palindromic Repeat
Cas: CRISPR-associated
TLC: Toxin-linked cryptic
RTX: Repeats in toxin
*ctx A*: Cholera toxin A subunit gene
*zot*: Zona occludens toxin gene
CDS: Coding sequence
VPI: Vibrio pathogenic island
KEGG: Kyoto Encyclopedia of Genes and Genomes
*rst*: Repeat sequence transcriptional regulator gene

## References

[1] Harris JB, LaRocque RC, Qadri F, Ryan ET, Calderwood SB (2012). Cholera. Lancet.

[2] Glass RI, Black RE. The Epidemiology of Cholera. In: Barua D, Greenough III WB, editors. Cholera. Current Topics in Infectious Disease. New York: Springer Science+Business Media, LLC.; 1992. p. 129–54.

[3] Miller CJ, Drasar BS, Feachem RG (1982). Cholera and estuarine salinity in Calcutta and London. Lancet.

[4] WHO. Cholera [web page]. 2022. https://www.who.int/news-room/fact-sheets/detail/cholera. Accessed 30 Mar.

[5] Clemens JD, Shin S, Sah BK, Sack DA, Plotkin SA, Orenstein WA, Offit PA (2013). Cholera vaccines. Vaccines.

[6] Barua D. History of Cholera. In: Barua D, Greenough III WB, editors. Cholera. Current Topics in Infectious Disease. New York: Springer Science+Business Media, LLC.; 1992. p. 1–36.

[7] Hu D, Liu B, Feng L, Ding P, Guo X, Wang M (2016). Origins of the current seventh cholera pandemic. Proc Natl Acad Sci U S A.

[8] Swaroop S, Pollitzer R (1955). Cholera studies. 2. World incidence. Bull World Health Organ.

[9] Kim EJ, Lee CH, Nair GB, Kim DW (2015). Whole-genome sequence comparisons reveal the evolution of Vibrio cholerae O1. Trends Microbiol.

[10] Tanizawa T. Sasebo Sengo Fukko no Ichi-Katei - Hikiage no Keiken -. Nagasaki-Kenritsu Daigaku Higashi-Asia Kenkyu-jyo “Higashi Asia Hyouron”. 2015 2015.03:147–60.

[11] Grant JR, Stothard P (2008). The CGView Server: a comparative genomics tool for circular genomes. Nucleic Acids Res.

[12] Prokka ST (2014). Rapid prokaryotic genome annotation. iSCB. Bioinformatics.

[13] Ohtsubo Y, Ikeda-Ohtsubo W, Nagata Y, Tsuda M (2008). GenomeMatcher: a graphical user interface for DNA sequence comparison. BMC Bioinformatics.

[14] Page AJ, Cummins CA, Hunt M, Wong VK, Reuter S, Holden MTG (2015). Roary: Rapid large-scale prokaryote pan genome analysis. Bioinformatics.

[15] Kozlov AM, Darriba D, Flouri T, Morel B, Stamatakis A (2019). RAxML-NG: a fast, scalable and user-friendly tool for maximum likelihood phylogenetic inference. Bioinformatics.

[16] Hadfield J, Croucher NJ, Goater RJ, Abudahab K, Aanensen DM, Harris SR (2018). Phandango: an interactive viewer for bacterial population genomics. Bioinformatics.

[17] Kanehisa M, Sato Y, Morishima K (2016). BlastKOALA and GhostKOALA: KEGG tools for functional characterization of genome and metagenome sequences. J Mol Biol.

[18] Grissa I, Vergnaud G, Pourcel C (2007). CRISPRFinder: a web tool to identify clustered regularly interspaced short palindromic repeats. Nucleic Acids Res.

[19] Mohapatra SS, Mantri CK, Turabe Fazil MH, Singh DV (2011). Vibrio cholerae O1 biotype El Tor strains isolated in 1992 from Varanasi, India harboured El Tor CTXPhi and classical ctxB on the chromosome-I and classical CTXPhi and classical ctxB on the chromosome-II. Environ Microbiol Rep.

[20] Pham TD, Nguyen TH, Iwashita H, Takemura T, Morita K, Yamashiro T (2018). Comparative analyses of CTX prophage region of Vibrio cholerae seventh pandemic wave 1 strains isolated in Asia. Microbiol Immunol.

[21] Rubin EJ, Lin W, Mekalanos JJ, Waldor MK (1998). Replication and integration of a Vibrio cholerae cryptic plasmid linked to the CTX prophage. Mol Microbiol.

[22] Lin W, Fullner KJ, Clayton R, Sexton JA, Rogers MB, Calia KE (1999). Identification of a vibrio cholerae RTX toxin gene cluster that is tightly linked to the cholera toxin prophage. Proc Natl Acad Sci U S A.

[23] Bakhshi B, Pourshafie MR, Navabakbar F, Tavakoli A (2008). Genomic organisation of the CTX element among toxigenic Vibrio cholerae isolates. Clin Microbiol Infect.

[24] Feng L, Reeves PR, Lan R, Ren Y, Gao C, Zhou Z (2008). A recalibrated molecular clock and independent origins for the cholera pandemic clones. PLoS ONE.

[25] Makarova KS, Koonin EV (2015). Annotation and classification of CRISPR-cas systems. Methods Mol Biol.

[26] Chun J, Grim CJ, Hasan NA, Lee JH, Choi SY, Haley BJ (2009). Comparative genomics reveals mechanism for short-term and long-term clonal transitions in pandemic Vibrio cholerae. Proc Natl Acad Sci U S A.

[27] Chin CS, Sorenson J, Harris JB, Robins WP, Charles RC, Jean-Charles RR (2011). The origin of the Haitian cholera outbreak strain. N Engl J Med.

[28] Mutreja A, Kim DW, Thomson NR, Connor TR, Lee JH, Kariuki S (2011). Evidence for several waves of global transmission in the seventh cholera pandemic. Nature.

[29] Murray PR, Rosenthal KS, Pfaller MA, Bacterial Metabolism and Genetics (2021). Medical microbiology.

[30] Murphy RA, Boyd EF (2008). Three pathogenicity islands of Vibrio cholerae can excise from the chromosome and form circular intermediates. J Bacteriol.

[31] Makarova KS, Wolf YI, Koonin EV (2013). Comparative genomics of defense systems in archaea and bacteria. Nucleic Acids Res.

[32] Box AM, McGuffie MJ, O'Hara BJ, Seed KD (2016). Functional analysis of bacteriophage immunity through a type I-E CRISPR-Cas system in vibrio cholerae and its application in bacteriophage genome engineering. J Bacteriol.

