# Supplementary material for: Whole-genome analysis of a Vibrio cholerae O1 biotype classical strain isolated in 1946 in Sasebo city, Nagasaki prefecture, from a returnee from the northeast part of China
Source: Trop Med Health. 2023 Feb 2;51:5. doi: 10.1186/s41182-023-00500-4 (PMC9893581; doi:10.1186/s41182-023-00500-4)
Supplement: Supplementary file 1 — Additional file 1: Fig. S1. The 94,615 bp fragment present in V. cholerae O1 biotype classical O395 but not in the Man 9 genome is depicted. Ninety-five CDSs are present in the fragment. Fig. S2. Kyoto Encyclopedia of Genes and Genomes (KEGG) analyses on the unique CDSs in two chromosomes carried by each strain Man9 and O395 were made. Thirty-five CDSs are unique to Man9, whereas 124 CDSs are unique to O395. Fig. S3. Profile of the Restriction Fragment Length Polymorphism (RFLP) with Southern blotting. The genome of O395 (Lane 1) and Man9 (Lane 2) were digested with BglI and visualized with zot- and ctxA—specific probes. M molecular marker. Fig. S4. PCR with the primer pair of TLC3F plus RTX5R and CIIF plus CIIR was applied on the genomic DNA extracted from Man9. An approx. 7.5 and 9.8 kb fragments were produced in separate reactions, estimated to contain the CTX prophage region on the Chr1, and the Chr2, respectively. The 9.8 kb product was further fragmented into three fragments; a 2.7 kb fragment with CIIF and rstBR primers; a 1 kb fragment with rstBF and rstRR primers; and a 6.6 kb fragment with rstRF and CIIR primers by PCR. The best-estimated configuration of the CTX prophage region, a CTXcla on the Chr1 and a CTXclaTrunc-CTXcla stretch on the Chr2, carried by Vibrio cholerae O1 biotype classical strain Man9 is shown. [file 41182_2023_500_MOESM1_ESM.pptx]

## Slide 1
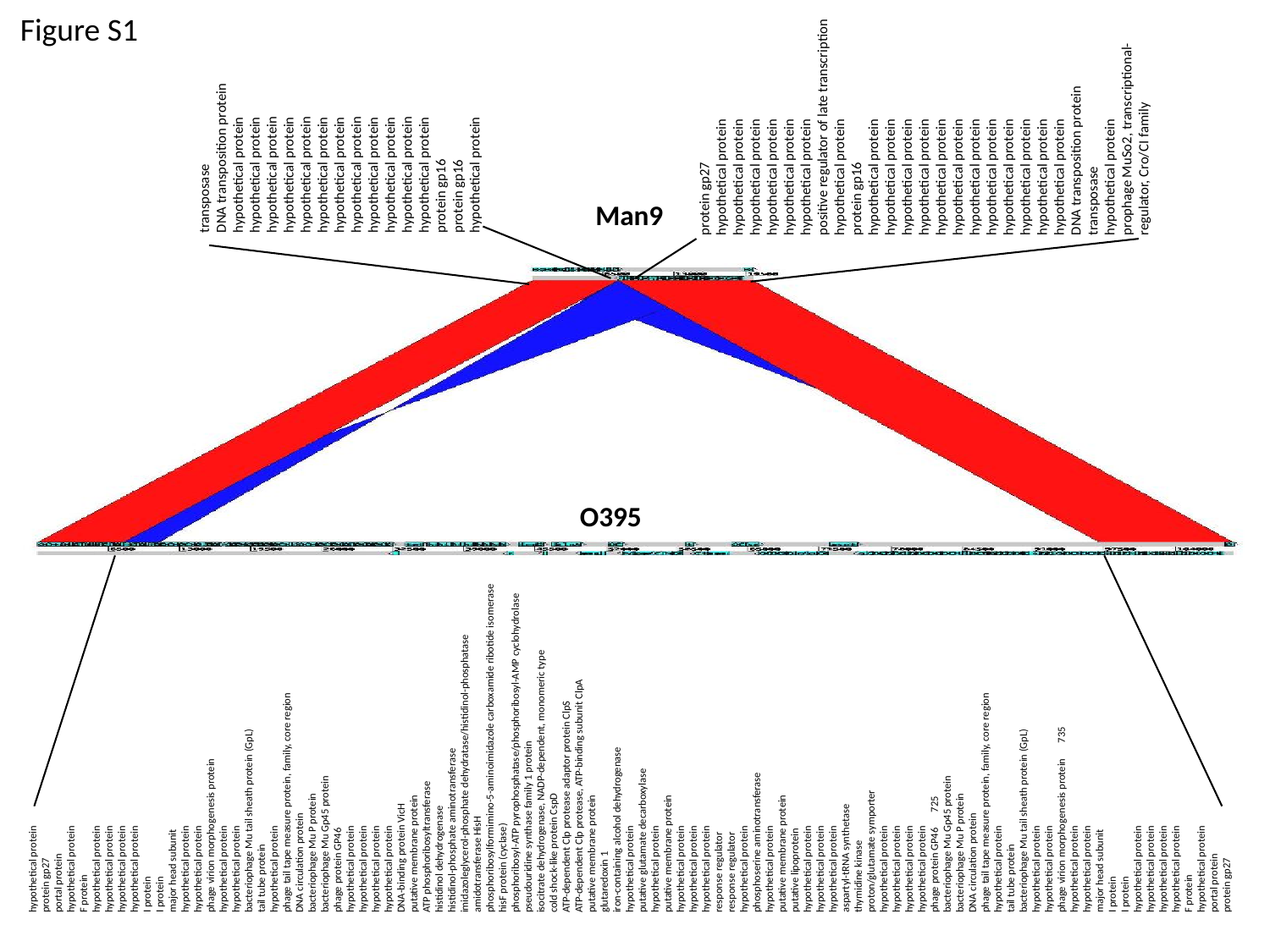

protein gp27
hypothetical protein
hypothetical protein
hypothetical protein
hypothetical protein
hypothetical protein
hypothetical protein
positive regulator of late transcription
hypothetical protein
protein gp16
hypothetical protein
hypothetical protein
hypothetical protein
hypothetical protein
hypothetical protein
hypothetical protein
hypothetical protein
hypothetical protein
hypothetical protein
hypothetical protein
hypothetical protein
hypothetical protein
DNA transposition protein
transposase
hypothetical protein
prophage MuSo2, transcriptional-
regulator, Cro/CI family
transposase
DNA transposition protein
hypothetical protein
hypothetical protein
hypothetical protein
hypothetical protein
hypothetical protein
hypothetical protein
hypothetical protein
hypothetical protein
hypothetical protein
hypothetical protein
hypothetical protein
hypothetical protein
protein gp16
protein gp16
hypothetical protein
Figure S1
hypothetical protein
protein gp27
portal protein
hypothetical protein
F protein
hypothetical protein
hypothetical protein
hypothetical protein
hypothetical protein
I protein
I protein
major head subunit
hypothetical protein
hypothetical protein
phage virion morphogenesis protein
hypothetical protein
hypothetical protein
bacteriophage Mu tail sheath protein (GpL)
tail tube protein
hypothetical protein
phage tail tape measure protein, family, core region
DNA circulation protein
bacteriophage Mu P protein
bacteriophage Mu Gp45 protein
phage protein GP46
hypothetical protein
hypothetical protein
hypothetical protein
hypothetical protein
DNA-binding protein VicH
putative membrane protein
ATP phosphoribosyltransferase
histidinol dehydrogenase
histidinol-phosphate aminotransferase
imidazoleglycerol-phosphate dehydratase/histidinol-phosphatase
amidotransferase HisH
phosphoribosylformimino-5-aminoimidazole carboxamide ribotide isomerase
hisF protein (cyclase)
phosphoribosyl-ATP pyrophosphatase/phosphoribosyl-AMP cyclohydrolase
pseudouridine synthase family 1 protein
isocitrate dehydrogenase, NADP-dependent, monomeric type
cold shock-like protein CspD
ATP-dependent Clp protease adaptor protein ClpS
ATP-dependent Clp protease, ATP-binding subunit ClpA
putative membrane protein
glutaredoxin 1
iron-containing alcohol dehydrogenase
hypothetical protein
putative glutamate decarboxylase
hypothetical protein
putative membrane protein
hypothetical protein
hypothetical protein
hypothetical protein
response regulator
response regulator
hypothetical protein
phosphoserine aminotransferase
hypothetical protein
putative membrane protein
putative lipoprotein
hypothetical protein
hypothetical protein
hypothetical protein
aspartyl-tRNA synthetase
thymidine kinase
proton/glutamate symporter
hypothetical protein
hypothetical protein
hypothetical protein
hypothetical protein
phage protein GP46　725
bacteriophage Mu Gp45 protein
bacteriophage Mu P protein
DNA circulation protein
phage tail tape measure protein, family, core region
hypothetical protein
tail tube protein
bacteriophage Mu tail sheath protein (GpL)
hypothetical protein
hypothetical protein
phage virion morphogenesis protein　735
hypothetical protein
hypothetical protein
major head subunit
I protein
I protein
hypothetical protein
hypothetical protein
hypothetical protein
hypothetical protein
F protein
hypothetical protein
portal protein
protein gp27
Man9
O395

## Slide 2
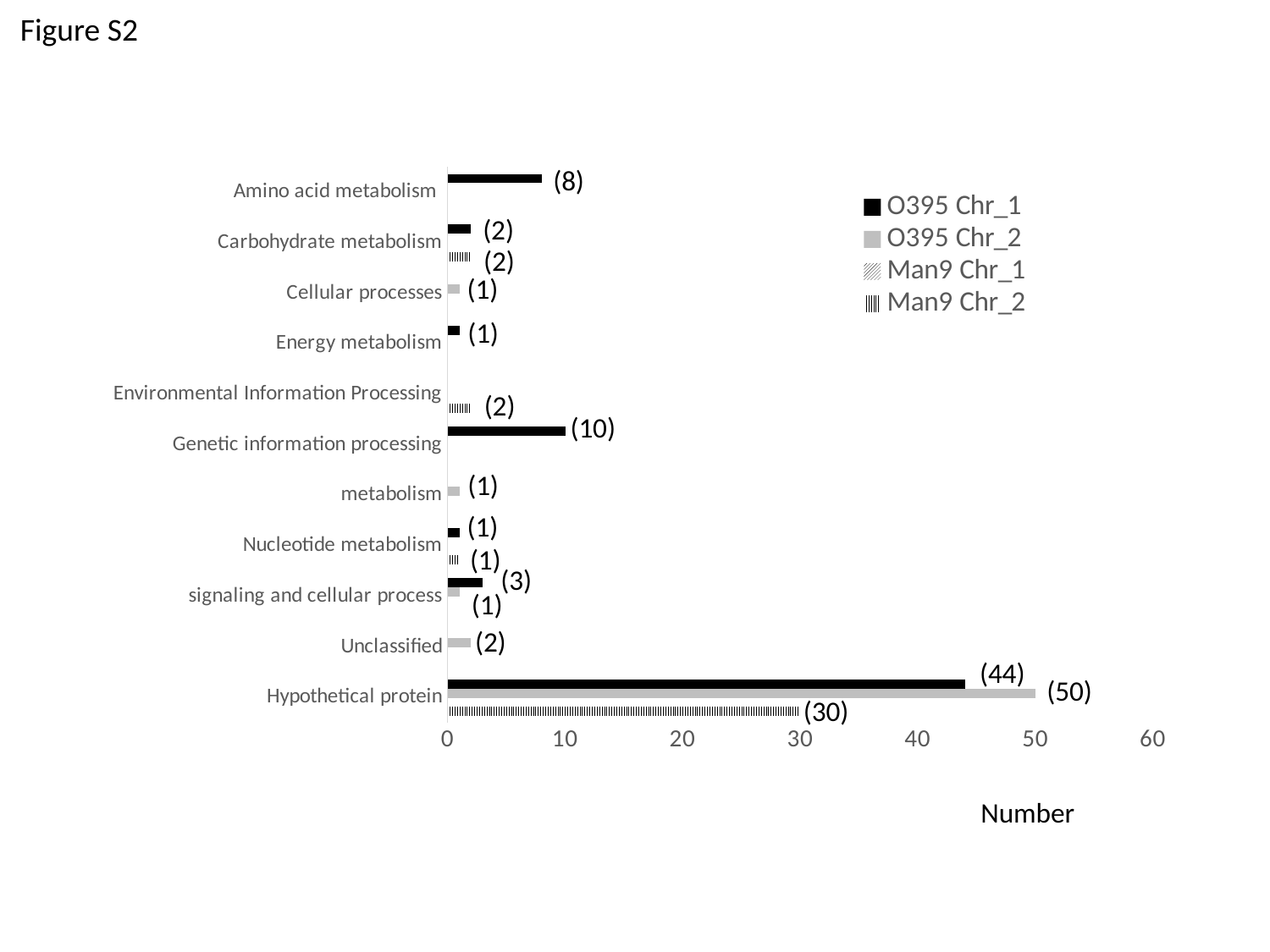

Figure S2
### Chart
| Category | Man9 Chr_2 | Man9 Chr_1 | O395 Chr_2 | O395 Chr_1 |
|---|---|---|---|---|
| Hypothetical protein | 30.0 | None | 50.0 | 44.0 |
| Unclassified | None | None | 2.0 | None |
| signaling and cellular process | None | None | 1.0 | 3.0 |
| Nucleotide metabolism | 1.0 | None | None | 1.0 |
| metabolism | None | None | 1.0 | None |
| Genetic information processing | None | None | None | 10.0 |
| Environmental Information Processing | 2.0 | None | None | None |
| Energy metabolism | None | None | None | 1.0 |
| Cellular processes | None | None | 1.0 | None |
| Carbohydrate metabolism | 2.0 | None | None | 2.0 |
| Amino acid metabolism | None | None | None | 8.0 |(8)
(2)
(2)
(1)
(1)
(2)
(10)
(1)
(1)
(1)
(3)
(1)
(2)
(44)
(50)
(30)
Number

## Slide 3
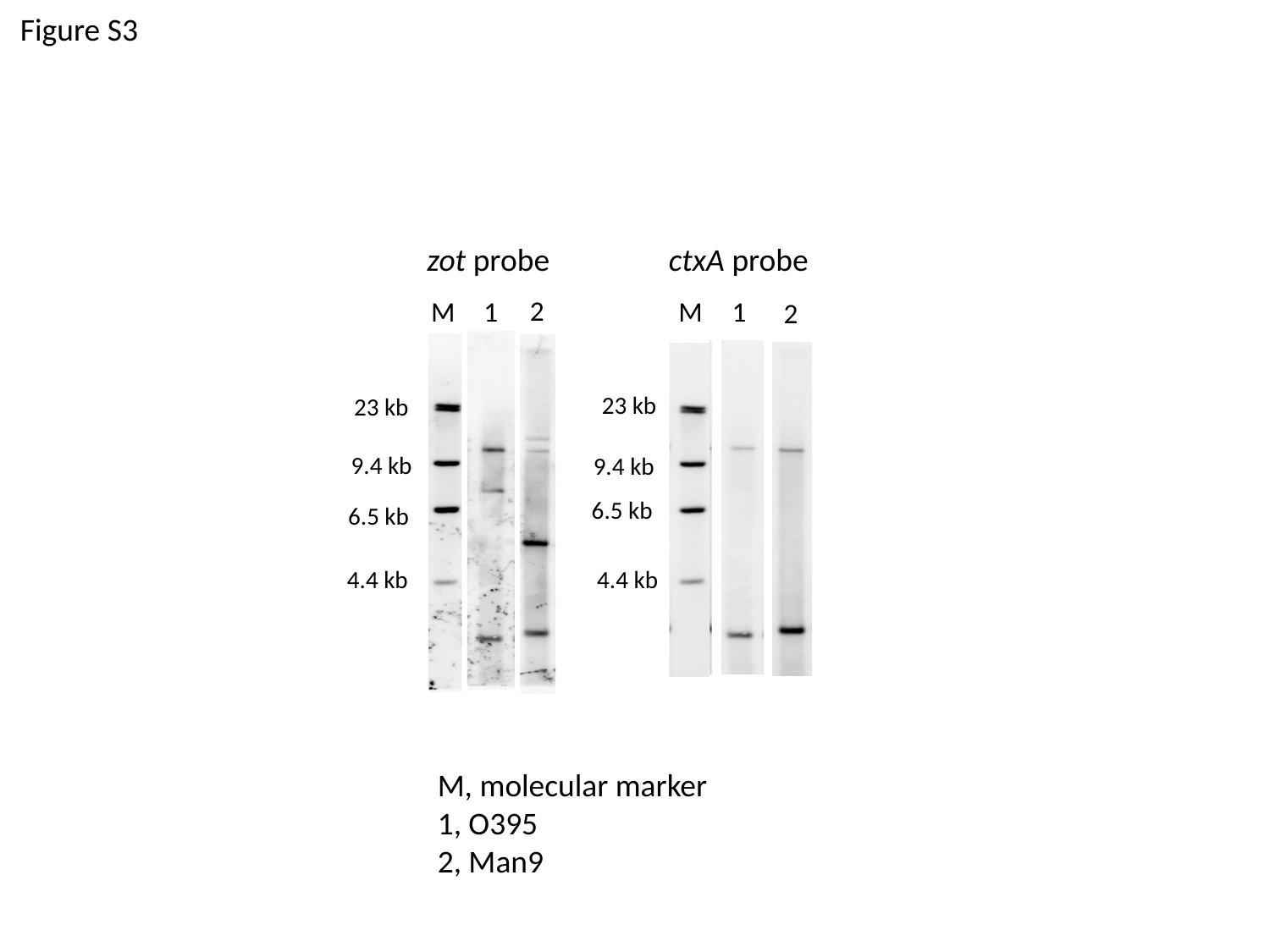

Figure S3
zot probe
ctxA probe
2
1
M
1
M
2
23 kb
23 kb
9.4 kb
9.4 kb
6.5 kb
6.5 kb
4.4 kb
4.4 kb
M, molecular marker
1, O395
2, Man9

## Slide 4
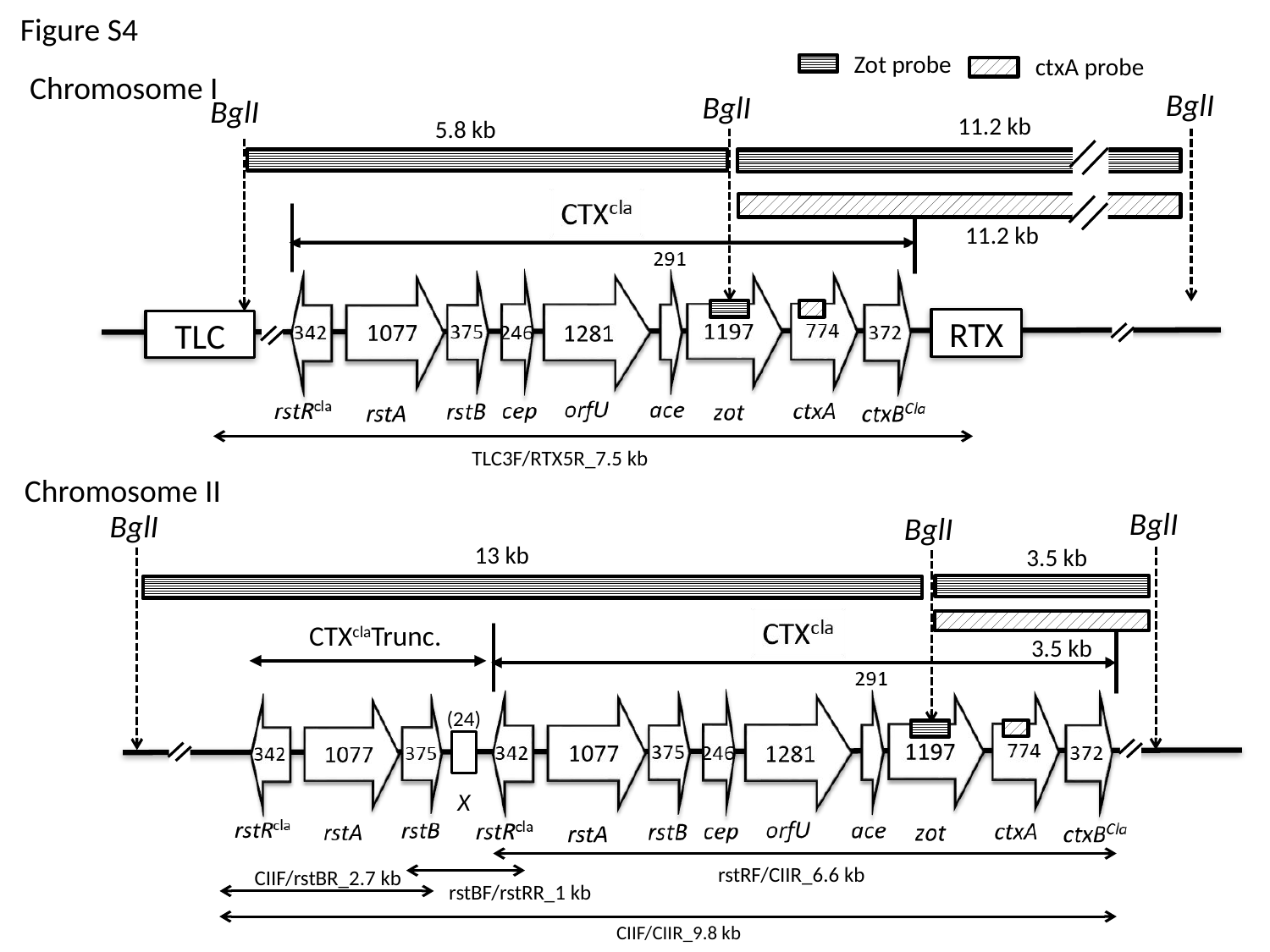

Figure S4
Zot probe
ctxA probe
Chromosome I
BglI
BglI
BglI
11.2 kb
5.8 kb
11.2 kb
RTX
TLC
TLC3F/RTX5R_7.5 kb
Chromosome II
BglI
BglI
BglI
13 kb
3.5 kb
CTXclaTrunc.
3.5 kb
(24)
X
rstRF/CIIR_6.6 kb
CIIF/rstBR_2.7 kb
rstBF/rstRR_1 kb
CIIF/CIIR_9.8 kb
