# Supplementary material for: Whole-genome analysis of a Vibrio cholerae O1 biotype classical strain isolated in 1946 in Sasebo city, Nagasaki prefecture, from a returnee from the northeast part of China
Source: Trop Med Health. 2023 Feb 2;51:5. doi: 10.1186/s41182-023-00500-4 (PMC9893581; doi:10.1186/s41182-023-00500-4)
Supplement: Supplementary file 2 — Additional file 2: Table S1. The List of V. cholerae O1 biotype classical strains (24 strains), biotype El Tor strains (11 strains), and a V. cholerae non O1, non O139 strain used to construct a phylogenetic tree. [file 41182_2023_500_MOESM2_ESM.pdf]

TableS 1

Strain name	Serogroup, Biotype	Accession Numbers	year	Country	Region	Sourse
1587	O12, Not applicable	SRX008805	1994	Peru	South_America	Clinical
A152	O1, El Tor	ERR018152	1991	Mozambique	Africa	Clinical
N16961	O1, El Tor	PRJNA606590	1975	Bangladesh	Asia	Clinical
MO10	O1, El Tor	PRJNA261682	1992	India	Asia	Clinical
VC150	O1, El Tor	SAMEA2006877	1992	Nepal	Asia	Clinical
V5	O1, El Tor	ERR018127	1989	India	Asia	Clinical
MJ_1236	O1, El Tor	PRJNA33555	1994	Bangladesh	Asia	Clinical
6196	O1, El Tor	ERR019293	2005	Kenya	Africa	Clinical
7686	O1, El Tor	ERR028075	2009	Kenya	Africa	Clinical
PRL64	O1, El Tor	ERR018195	1992	India	Asia	Clinical
4662	O1, El Tor	ERR025373	2001	Bangladesh	Asia	Clinical
HFU_02	O1, El Tor	CP043554.1, CP043556.1	2010	Haiti	Calibbian	Clinical
A46	O1, classical	ERR018132	1964	unknown	unknown	Clinical
A51	O1, classical	ERR018141	1949	Egypt	Africa	Clinical
A59	O1, classical	ERR018139	1970	India	Asia	Clinical
A68	O1, classical	ERR018135	1949	Egypt	Africa	Clinical
RC27	O1, classical	PRJNA40505	1991	Indonesia	Asia	Clinical
A111	O1, classical	ERR018155	1990	unknown	unknown	Clinical
Man9	O1, classical	This study	1946	Japan	Asia	Clinical
A50	O1, classical	ERR018142	1953	Bangladesh	Asia	Clinical
A49	O1, classical	ERR018134	1952	unknown	unknown	Clinical
A61	O1, classical	ERR018137	1970	India	Asia	Clinical
A57	O1, classical	ERR018140	1980	India	Asia	Clinical
A60	O1, classical	ERR018138	1958	Thailand	Asia	Clinical
O395	O1, classical	PRJNA293904	1965	India	Asia	Clinical
A66	O1, classical	ERR018136	1962	Bangladesh	Asia	Clinical
A103	O1, classical	ERR018145	1990	unknown	unknown	Clinical
A76	O1, classical	ERR018143	1982	Bangladesh	Asia	Clinical
A389	O1, classical	ERR018178	1987	Bangladesh	Asia	Clinical
A279	O1, classical	ERR018173	1990	Sweden	Europe	Clinical
GP8	O1, classical	ERR018129	1970	India	Asia	Clinical
A70	O1, classical	ERR018133	1969	Bangladesh	Asia	Clinical
GP16	O1, classical	ERR018184	1971	India	Asia	Clinical
VC201	O1, classical	ERR125427-ERR125431	1985	Pakistan	Asia	Clinical
VC200	O1, classical	ERR125425-ERR125426	1985	Pakistan	Asia	Clinical
VC214	O1, classical	ERR125441-ERR125445	unknown	Pakistan	Asia	Clinical

Strain name	Accession	scafold	year	Country	Region	Sourse
V52	PRJNA224116	V52.fasta	1968	Sudan	Africa	Clinical
A46	ERR018132	scaf_.fasta	1964	unknown	unknown	Clinical
A51	ERR018141	scaf_A51ERR018141.fasta	1949	Egypt	Africa	Clinical
RC27	PRJNA40505	RC27.fasta	1991	Indonesia	Asia	Clinical
A111	ERR018155	scaf_.fasta	1990	unknown	unknown	Clinical
Man9	This study	This study	This study	This study	This study	This study
GP8	ERR018129	scaf_.fasta	1970	India	Asia	Clinical
A70	ERR018133	scaf_ERR018133.fasta	1969	Bangladesh	Asia	Clinical
VC200	ERR125425-ERR125426	scaf_SAMEA2007293.fasta	1986	Pakistan	Asia	Clinical
VC214	ERR125441-ERR125445	scaf_SAMEA1972393.fasta	unknown	Pakistan	Asia	Clinical
VC201	ERR125427-ERR125431	scaf_SAMEA2007294.fasta	1986	Pakistan	Asia	Clinical
GP16	ERR018184	scaf_ERR018184.fasta	1971	India	Asia	Clinical
A389	ERR018178	scaf_ERR018178.fasta	1987	Bangladesh	Asia	Clinical
A103	ERR018145	scaf_ERR018145.fasta	1990	unknown	unknown	Clinical
A66	ERR018136	scaf_ERR018136.fasta	1962	Bangladesh	Asia	Clinical
A76	ERR018143	scaf_ERR018143.fasta	1982	Bangladesh	Asia	Clinical
A279	ERR018173	scaf_A279ERR018173.fasta	1990	Sweden	Europe	Clinical
O395	PRJNA293904	O395.fasta	1965	India	Asia	Clinical
A60	ERR018138	scaf_A60ERR018138.fasta	1958	Thailand	Asia	Clinical
A57	ERR018140	scaf_ERR018140.fasta	1980	India	Asia	Clinical
A61	ERR018137	scaf_ERR018137.fasta	1970	India	Asia	Clinical
A49	ERR018134	scaf_ERR018134.fasta	1962	unknown	unknown	Clinical
A50	ERR018142	scaf_ERR018142.fasta	1963	Bangladesh	Asia	Clinical
A59	ERR018139	scaf_ERR018139.fasta	1970	India	Asia	Clinical
A68	ERR018135	scaf_ERR018135.fasta	1949	Egypt	Africa	Clinical
7686	ERR028075	scaf_ERR028075.fasta	2009	Kenya	Africa	Clinical
6196	ERR019293	scaf_ERR019293.fasta	2005	Kenya	Africa	Clinical
PRL64	ERR018195	scaf_PRL64ERR018195.fasta	1992	India	Asia	Clinical
4662	ERR025373	scaf_ERR025373.fasta	2001	Bangladesh	Asia	Clinical
V5	ERR018127	scaf_V5ERR018127.fasta	1989	India	Asia	Clinical
MO10	PRJNA261682	MO10.fasta	1992	India	Asia	Clinical
MJ_1236	PRJNA33555	MJ-1236.fasta	1994	Bangladesh	Asia	Clinical
A152	ERR018152	scaf_ERR018152.fasta	1991	Mozambique	Africa	Clinical
A201	ERR018167	scaf_A201ERR018167.fasta	1992	Argentina	South_America	Clinical
N16961	PRJNA606590	N16961.fasta	1975	Bangladesh	Asia	Clinical
MAK757	SRX002426	MAK-757.fasta	1937	Indonesia	Asia	Clinical
MZO_3	PRJNA18261	MZO-3.fasta	2001	Bangladesh	Asia	Clinical
1587	SRX008805	1587.fasta	1994	Peru	South_America	Clinical

Supplemental Table 2

Strain name	Data access	scafold	year	Country	Region	Sourse
V52	PRJNA224116	V52.fasta	1968	Sudan	Africa	Clinical
A46	ERR018132	scaf_.fasta	1964	unknown	unknown	Clinical
A51	ERR018141	scaf_A51ERR018141.fasta	1949	Egypt	Africa	Clinical
RC27	PRJNA40505	RC27.fasta	1991	Indonesia	Asia	Clinical
A111	ERR018155	scaf_.fasta	1990	unknown	unknown	Clinical
Man9	This study	This study	This study	This study	This study	This study
GP8	ERR018129	scaf_.fasta	1970	India	Asia	Clinical
A70	ERR018133	scaf_ERR018133.fasta	1969	Bangladesh	Asia	Clinical
VC200	ERR125425-ERR125426	scaf_SAMEA2007293.fasta	1986	Pakistan	Asia	Clinical
VC214	ERR125441-ERR125445	scaf_SAMEA1972393.fasta	unknown	Pakistan	Asia	Clinical
VC201	ERR125427-ERR125431	scaf_SAMEA2007294.fasta	1986	Pakistan	Asia	Clinical
GP16	ERR018184	scaf_ERR018184.fasta	1971	India	Asia	Clinical
A389	ERR018178	scaf_ERR018178.fasta	1987	Bangladesh	Asia	Clinical
A103	ERR018145	scaf_ERR018145.fasta	1990	unknown	unknown	Clinical
A66	ERR018136	scaf_ERR018136.fasta	1962	Bangladesh	Asia	Clinical
A76	ERR018143	scaf_ERR018143.fasta	1982	Bangladesh	Asia	Clinical
A279	ERR018173	scaf_A279ERR018173.fasta	1990	Sweden	Europe	Clinical
O395	PRJNA293904	O395.fasta	1965	India	Asia	Clinical
A60	ERR018138	scaf_A60ERR018138.fasta	1958	Thailand	Asia	Clinical
A57	ERR018140	scaf_ERR018140.fasta	1980	India	Asia	Clinical
A61	ERR018137	scaf_ERR018137.fasta	1970	India	Asia	Clinical
A49	ERR018134	scaf_ERR018134.fasta	1962	unknown	unknown	Clinical
A50	ERR018142	scaf_ERR018142.fasta	1963	Bangladesh	Asia	Clinical
A59	ERR018139	scaf_ERR018139.fasta	1970	India	Asia	Clinical
A68	ERR018135	scaf_ERR018135.fasta	1949	Egypt	Africa	Clinical
7686	ERR028075	scaf_ERR028075.fasta	2009	Kenya	Africa	Clinical
6196	ERR019293	scaf_ERR019293.fasta	2005	Kenya	Africa	Clinical
PRL64	ERR018195	scaf_PRL64ERR018195.fasta	1992	India	Asia	Clinical
4662	ERR025373	scaf_ERR025373.fasta	2001	Bangladesh	Asia	Clinical
V5	ERR018127	scaf_V5ERR018127.fasta	1989	India	Asia	Clinical
MO10	PRJNA261682	MO10.fasta	1992	India	Asia	Clinical
MJ_1236	PRJNA33555	MJ-1236.fasta	1994	Bangladesh	Asia	Clinical
A152	ERR018152	scaf_ERR018152.fasta	1991	Mozambique	Africa	Clinical
N16961	PRJNA606590	N16961.fasta	1975	Bangladesh	Asia	Clinical
VC150	SAMEA2006877		1992	Nepal	Asia	Clinical
HFU-02	CP043554.1, CP043556.1		1992	Haiti	Calibbian	Clinical
MAK757	SRX002426	MAK-757.fasta	1937	Indonesia	Asia	Clinical
MZO_3	PRJNA18261	MZO-3.fasta	2001	Bangladesh	Asia	Clinical
1587	SRX008805	1587.fasta	1994	Peru	South_America	Clinical
