# Supplementary material for: Whole-genome analysis of a Vibrio cholerae O1 biotype classical strain isolated in 1946 in Sasebo city, Nagasaki prefecture, from a returnee from the northeast part of China
Source: Trop Med Health. 2023 Feb 2;51:5. doi: 10.1186/s41182-023-00500-4 (PMC9893581; doi:10.1186/s41182-023-00500-4)
Supplement: Supplementary file 3 — Additional file 3: Table S2. The presence or the absence of the CTX prophage-associated genes and their positional relationship on the Chr1 and the Chr2 was confirmed by the PCR profile using a series of primers [19]. [file 41182_2023_500_MOESM3_ESM.pdf]

Table S2

| Strain | Size of fragments, kb *BglI* digestion *zot* probe | Size of fragments, kb *BglI* digestion *ctxA* probe | PCR profile TLC3F/ rstAR | TLC3F/ rstC2 | TLC3F/ rstRclaR | TLC3F/ rstRETR | ctxAF/ rtx5R | ctxBF/ ctxBR | rstRclaF/ rstRclaR | rstRETF/ rstRETR | CIIF/ CIIR | CIIF/ rstAR | CIIF/ rstC2 | CIIF/ rstRclaR | CIIF/ rstRETR | ctxAF/ CIIR |
|---|---|---|---|---|---|---|---|---|---|---|---|---|---|---|---|---|
| Man9 | 13; 11.2; 5.8; 3.5 | 11.2; 3.5 | P | N | P | N | P | P | P | N | N | P | N | P | N | P |
| O395 | 11.2; 7.6; 3.5 | 11.2; 3.5 | P | N | P | N | P | P | P | N | N | P | N | P | N | P |
